# Untangling the relationship between bempedoic acid and gout: results from a systematic literature review

**DOI:** 10.3389/fcvm.2023.1234601

**Published:** 2023-10-25

**Authors:** Alessia Alunno, Francesco Carubbi, Elena Campanozzi, Federico Bellisario, Jan W. Schoones, Francesco Maria Mariani, Evy Di Ruscio, Piera Altieri, Claudio Ferri

**Affiliations:** ^1^ Department of Clinical Medicine, Life, Health & Environmental Sciences, Internal Medicine and Nephrology Division, ASL1 Avezzano-Sulmona-L'Aquila, San Salvatore Hospital, University of L’Aquila, L'Aquila, Italy; ^2^ Directorate of Research Policy, Leiden University Medical Center, Leiden, Netherlands

**Keywords:** bempedoic acid, uric acid, gout, low density lipoproteins, cholesterol

## Abstract

**Background:**

Bempedoic acid (BA) is a small-molecule first-in-class of inhibitor of ATP citrate lyase that significantly lowers low-density lipoproteins cholesterol (LDL-c) in statin-intolerant and inadequate responders. Increased serum uric acid (SUA) levels and gout incidence have been described in BA-treated patients. The aim of this systematic review was to investigate the safety of BA regarding SUA levels and gout in randomised controlled trials (RCTs).

**Methods:**

A search on 7 databases was performed from inception to May 4, 2023. RCTs of BA monotherapy or combination with other lipid-lowering treatment (LLT) in patients with increased LDL-c were included. Dual data extraction was performed with disagreements resolved through consensus. Due to the methodological purpose of this review risk-of-bias assessment of studies was not performed.

**Results:**

6 Phase 3 RCTs (*N* = 17,975 patients of which 9,635 received BA) 9 Phase 2 RCTs (*N* = 362 patients of which 170 received BA) and an open-label extension of a Phase 3 RCT were included. Gout and/or hyperuricemia were not mentioned as exclusion criteria, previous/current use of urate-lowering therapies (ULT) and/or colchicine and/or dietary patterns were not reported. Phase 3 RCTs: 2 studies specified the number of patients experiencing hyperuricemia over the study period (BA: 4.9%–11%; placebo: 1.9%–5.6%) and the effect size was significant only in 1 study (OR = 2.0, 95% CI 1.8–2.3). Four RCTs reported a higher incidence of gout in the BA arm however, when we calculated the effect size, it was small and often not significant. Two studies reported 0 cases of gout. The paucity of information about SUA levels at baseline and/or at the end of follow-up do not allow us to quantify the effect sizes for BA-induced SUA elevation. Data on gout from Phase 2 RCTs is scant.

**Conclusions:**

Data from phase 2 and 3 RCTs do not allow for confirming a clear association between BA and gout. It is conceivable that a careful assessment of SUA levels/history of gout at baseline and the concomitant use of urate-lowering agents may be instrumental to minimise the risk of new-onset gout/gout flares in patients treated with BA.

## 1. Introduction

Lifestyle modifications are the first line approach for lipid management whereas statins are the mainstay of treatment in those who do not achieve the target with non-pharmacological approaches (1). Statin treatment is safe and the benefit for CV prevention goes beyond the mere reduction of cholesterol encompassing their anti-inflammatory, antithrombotic and antioxidant actions (2). However, safety concerns particularly related to muscle side effect are among the most significant determinants of poor statin adherence and therapy withdrawal [statin intolerant (INT) patients] (3). On the contrary, a considerable number of patients may still have high cholesterol level despite the maximum tolerated stating dose [statin inadequate responder (IR) patients] and require additional lipid-lowering therapies (LLT). Randomised controlled trials (RCTs) evaluating combination of statins with ezetimibe demonstrated that this combination is adequate in patients with low to moderate risk but still at least one-third patients with high or very high CV risk will require the addition of a third compound to achieve the LDL-c target. The approval of proprotein convertase subtilisin/kexin type 9 (PCSK9) inhibitors was a milestone in the history of lipid management, however it posed economic issues with regard to the broad use in the large population of statin-IR and statin-INT (4). Therefore, the use of new cost-effective LLTs associated with reduced muscular side effects can definitely improve the success of treatment.

Bempedoic acid (BA), formerly ETC-1002, is a small-molecule first-in-class of inhibitors of ATP citrate lyase (5). In 2016 the key papers by Pinkosky et al. demonstrated that BA led to low density lipoprotein (LDL) receptor upregulation, LDL-cholesterol (c) decrease, attenuation of atherosclerosis, reduction of hepatic lipids and body weight and improved glycaemic control (6, 7). BA effects on LDL-c can be detected when given both alone and as add-on therapy to other LLTs and it also shows anti-inflammatory effects by decreasing high-sensitivity C-reactive protein. BA seems a promising approach for optimal lipid management, however its effect on serum uric acid (SUA) levels raised safety concerns. BA is known to be associated with modest SUA elevation and the putative mechanism is the competition between the BA glucuronide metabolite and UA for the same renal transporters involved in the excretion of these compounds (8). SUA is a recognised CV risk factor (9) and prognostic SUA thresholds have been identified with regard to CV mortality and CV events such as cerebrovascular diseases (10–12). Therefore, not only it seems contradictory to use a drug able to control one CV risk factor while triggering another one, but the evidence of a higher incidence of gout induced by BA in the recently published CLEAR Outcome large RCT (13) added a layer of complexity. In fact, since the target population of BA treatment is often multimorbid and shows high to very CV risk, is it imperative to clarify the safety profile of this drug to avoid overestimation of an adverse event whose incidence may be minimised or even prevented. On this basis, we aimed to explore in detail the available data on SUA and gout from RCTs assessing BA and clarify their strength and clinical relevance.

## 2. Methods

A systematic literature search was performed on PubMed, Embase, Web of Science, Cochrane Library, Emcare, Academic Search Premier and Google Scholar from inception to 4 May 2023 according to the Preferred Reporting Items for Systematic Reviews and Meta-analyses (PRISMA) guidelines (Supplementary Table S1). The search strategy was developed by an expert librarian (JWS) based on the Population/Intervention/Comparator/Outcome (PICO) framework (Supplementary Text S1). Title and abstract screening, as well as the article full-text review, was conducted in duplicate independently by two authors (EC and FB) with any disagreements resolved by discussion. The following inclusion criteria were used: English language Phase 2 and Phase 3 RCT were included if they enrolled patients undergoing treatment with BA regardless of the study outcomes. Data extraction was performed independently in duplicate using a pilot-tested data extraction form. Data pertaining to the article's identifying information, methods, population, interventions, efficacy and safety outcomes were extracted. The risk of bias of included articles was not assessed as we were mostly interested in reviewing methodological aspects of the studies. Likewise, meta-analysis and related assessments (e.g., Cochran's Q for heterogeneity) were not performed. However, to facilitate comparison of studies, effect size (unadjusted odds ratios (OR) or standardised mean differences and 95% confidence intervals (CI)) was computed using the data available in the included articles.

## 3. Results

A total of 745 references were retrieved (317 after deduplication) of which 23 were eligible for full text assessment and 16 were included in the review (Supplementary Figure S1). Six of the 16 articles were phase 3 RCTs (13–18), 1 article was the open-label extension (OLE) of one RCT (19) and 9 articles were phase 2 trials.

### 3.1. Phase 3 RCTs

Of the 6 phase 3 RCTs, 5 belonged to the CLEAR (Cholesterol Lowering via Bempedoic acid, an ACL-Inhibiting Regimen) trial program (13–17). All 6 RCTs recruited a total of 17,975 patients of which 9’635 received BA (Supplementary Table S2). In 5 studies the primary endpoint was the percentage of LDL-c change at 12 weeks (W) and the follow-up period ranged between 12 and 52 W (14–18). In the most recent and largest RCT, the CLEAR Outcome, the primary endpoint was a four-component composite of major adverse CV events, defined as death from CV causes, nonfatal myocardial infarction, nonfatal stroke, or coronary revascularization, as assessed in a time-to-first-event analysis with a total follow up time of 60 months (13). In studies with follow up of 52 W or longer (13, 14, 16), it was possible to adjust ongoing LLT from 24 W onwards at clinician discretion in inadequate responders, namely those with LDL-c >170 mg/dl and LDL-c increased by at least 25% from baseline. Gout and/or hyperuricemia were not mentioned as exclusion criteria, previous/current use of urate-lowering therapies (ULT) and/or colchicine as well as specific dietary patterns were not reported either. With regard to SUA, baseline levels in the full study cohort were reported only in 2 studies (14, 17) and only one of these (17) also reported the SUA levels at the end of follow up but only in the BA treatment arm (Table 1).

**Table 1:** Summary of data on serum uric acid and gout in the phase 3 randomised controlled trials investigating bempedoic acid.

Study name or RCT *N* Year Reference	Follow up (weeks)	Intervention, *N* males *N* (%)	*N* (%) patients with gout over study period	*N* (%) patients with hyperuricemia over study period	SUA mean (SD) (mg/dl)		Δ SUA mean (SD) (mg/dl)	Previous history of gout or hyperuricemia
		Comparator, *N* males *N* (%)			Baseline	End of follow up		
CLEAR Tranquility (2018) (17)	12	BA + EZE: 181 72 (40)	0 (0)	NR^a^	5.8 (1.4)	6.3 (1.5)	NR	SUA>ULN at baseline in 13/16 patients who developed hyperuricemia
		PBO: 88 32 (36)	0 (0)		NR	NR		
CLEAR Serenity (2019) (15)	24	BA: 234 101 (43)	NR (1.7)	NR	NR	NR	0.7–0.9	NR
		PBO: 111 61 (45)	NR (0.9)				0 to −0.12	
CLEAR Harmony (2019) (16)	52	BA: 1,488 1,099 (74)	18 (1.2)	NR	NR	NR	0.73 (1.1)	NR
		PBO: 742 529 (71)	2 (0.3)				−0.06 (0.9)	
CLEAR Wisdom (2019) (14)	52	BA: 522 328 (63)	11 (2.1)	22 (4.2)	5.95 (1.5)	NR	0.6 (1.2)	Gout 5/11 and hyperuricemia 3/11 in the 11 patients in the BA group developing gout over the study period
		PBO: 257 168 (65)	2 (0.8)	5 (1.9)	5.97 (1.4)		0.1 (1.1)	
NCT03337308 (2020) (18)	12	BA + EZE: 108 NR	0 (0)	NR^a^	NR	NR	0.42 (0.76)	NR
		BA: 110 NR	0 (0)				0.59 (0.75)	
		EZE: 109 NR	0 (0)				0.03 (0.52)	
		PBO: 55 NR	0 (0)				−0.1 (0.54)	
CLEAR Outcomes (2023) (13)	240	BA: 6,992 3,331 (52)	215 (3.1)	763 (10.9)	NR	NR	0.76 (1.2)	NR
		PBO: 6,978 3,599 (51)	143 (2.1)	393 (5.6)			−0.03 (1.0)	

RCT, randomised controlled trial; BA, bempedoic acid; EZE, ezetimibe; PBO, placebo; SUA, serum uric acid; SD, standard deviation; N, number; NR, not reported; ULN, upper level of normal.

^a^ The authors reported the number of patients developing an increase of SUA but they did not not specify if this increase led to values above the threshold for hyperuricemia.

The other RCTs reported neither baseline nor end of follow up SUA values. Five RCTs reported the mean SUA change from baseline that ranged between 0.4 and 0.9 mg/dl in the BA arm and −0.06 and 0.1 mg/dl in the placebo arm (13–16, 18). Two RCTs specified the number of patients experiencing hyperuricemia over the study period that ranged between 4.9% and 11% in the BA-arm and between 1.9 and 5.6% in the placebo arm (13, 14). The CLEAR Tranquility and NCT03337308 studies reported the number of patients experiencing an increase of SUA but the Authors did not specify if this increase was classified as hyperuricemia (17, 18).

**Figure 1:**
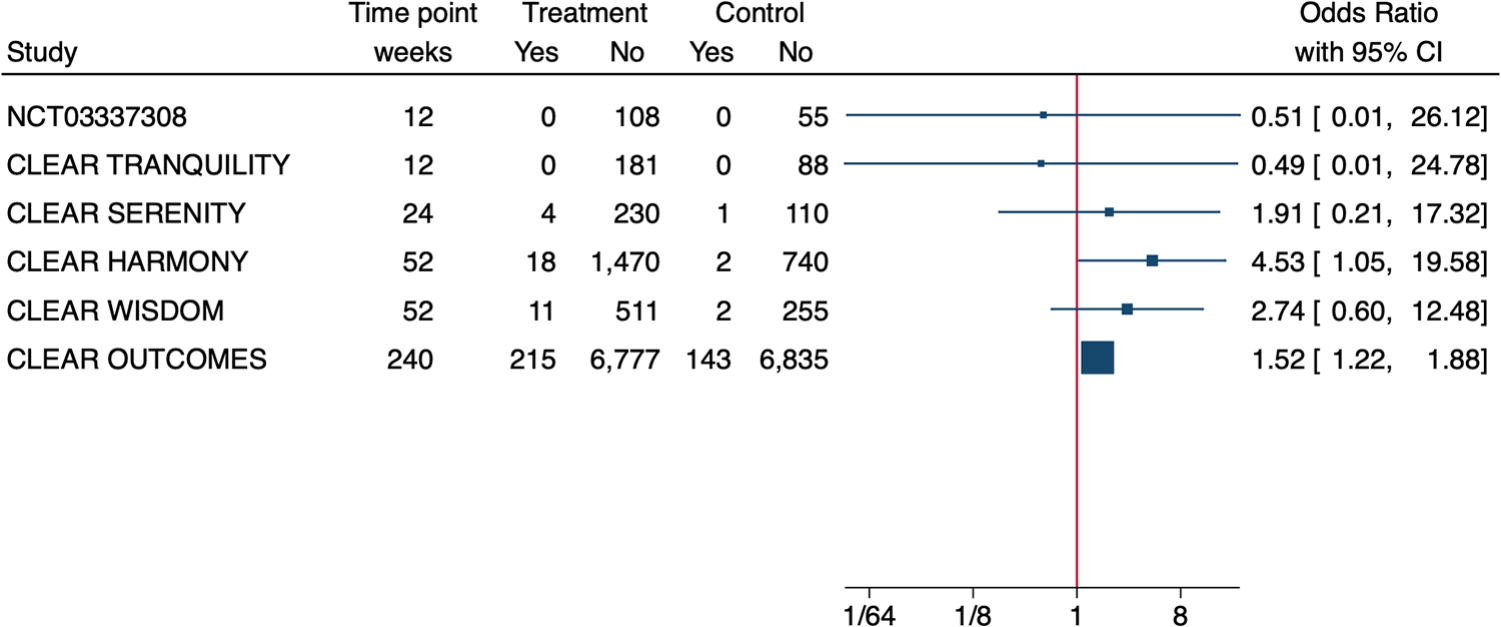
Effect size of bempedoic acid on the incidence of gout in phase 3 randomised controlled trials. Due to the heterogeneity of patient populations and follow-up time a meta-analysis was not performed.

The effect size for hyperuricemia was significant only in the CLEAR Outcome (OR = 2.0, 95% CI 1.8–2.3) (13) but not in the CLEAR Wisdom (OR = 2.2 95% CI = 0.8–5.9) (14). In addition, the Authors of the CLEAR Tranquility study specified that in the 81% of patients whose SUA levels increased in either study arm over the study period, they were above upper limit of normal (ULN) at baseline (17). The OLE of the CLEAR Harmony study (19) provided a nice graphical overview of SUA levels during the study treatment showing that the average SUA levels at entry in the parent study were slightly above 6 mg/dl, it increased over the first 12 W of treatment and then it remained stable around 7 mg/dl over the follow up of 130 W (BA since randomization in the parent study, BA➝BA). In patients treated with placebo in the parent study that were switched to BA at OLE enrolment (PBO➝BA), SUA rapidly increased over the first 12 W and remained stable over the 78 W of follow up with concentrations comparable to those of the BA➝BA population. With regard to overt gout, 4 RCTs (13–16) reported a higher incidence in the BA-treated patients (Table 1) however, when we calculated the effect size, we noticed that it was small and often not significant (Figure 1).

In addition, the CLEAR Tranquility and the NCT03337308 study reported 0 cases of gout across all treatment arms over the 12W of follow up (17, 18). The OLE of the CLEAR Harmony study (19) reported an incidence of gout of 2.5% in the BA➝BA group and of 2.8% in the PBO➝BA group at the end of follow-up. Previous history of hyperuricemia/gout in patients developing gout during the study period was described only in the CLEAR Wisdom study (14). In particular, 91% of the patients developing gout in the BA treatment arm, had SUA levels >ULN at the time of enrolment, 45% had history of gout and 27% had a history of hyperuricemia before study enrolment. No data about history of gout in the full study cohorts was provided.

### 3.2. Phase 2 RCTs

Of the 9 phase 2 studies retrieved by the search, only 6 reported information on SUA levels and/or gout over the study period and will be discussed here (20–25). (Supplementary Table S3 and Table 2).

**Table 2:** Summary of data on serum uric acid and gout in the phase 2 randomised controlled trials investigating bempedoic acid.

Author, year	Follow up (weeks)	Intervention, *N* patients	*N* (%) patients with gout over study period	*N* (%) patients with hyperuricemia over study period	Δ SUA Mean (SD) (mg/dl)
RCT *N*, ref		Comparator, *N* patients			
Ballantyne CM, 2013	12	BA 40 mg *N* = 45	Gout mentioned among adverse events, no incidence provided	In BA-treated patients mean SUA increased by 7% to 16%.	NR
		BA 80 mg *N* = 44			
		BA 120 mg *N* = 44			
NCT01262638 (25)		PBO *N* = 44			
Gutierrez MJ, 2014	4	BA 80 mg➝ 120 mg *N* = 30	NR	In BA-treated patients mild to moderate mean SUA increase	NR
NCT01607294 (24)		PBO *N* = 30			
Thompson PD, 2015	8	BA 60 mg➝ 120 mg➝ 180mg➝ 240 mg *N* = 37	NR	NR	1.2 (0.7)
NCT01751984 (23)		PBO *N* = 19			0.08 (0.9)
Lalwani ND, 2019	4	BA 180 mg *N* = 45	0 (0)	NR	NR
NCT02659397 (21)		PBO *N* = 23	1 (4)		
Bays HE, 2021	12	BA 180 mg + EZE 10 mg *N* = 60	0 (0)	No patient reported increased SUA	NA
NCT03531905 (22)		EZE 10 mg *N* = 60	0 (0)		
		PBO *N* = 59			
Rubino J, 2021	8	BA 180 mg *N* = 28	1 (3.6)	NR	0. 56 (0.77)
NCT03193047 (20)		PBO *N* = 31	0 (0)		−0. 03 (0.56)

RCT, randomised controlled trial; BA, bempedoic acid; EZE, ezetimibe; PBO, placebo; SUA, serum uric acid; SD, standard deviation; N, number; NR, not reported; ULN, upper level of normal.

A total of 362 patients were enrolled of which 170 received BA for a period ranging between 4 and 12 W. The 3 more recent studies (20–22) used a fixed dose of BA (180 mg/day) like the above described RCTs, whereas the studies published before 2019 used either an increasing dose (23, 24) or more than one treatment arm with different doses (25). Furthermore, all studies included a washout period ranging between 4 and 6 weeks when ongoing LLT were discontinued before being randomised to BA or other treatment arms. Another important aspect pertains to combination therapy since in one study OL atorvastatin 80 mg was started during the washout period (21), whereas in another study the washout period was followed by OL evolocumab for 3 months before being randomised to BA or other treatment arms (20). Gout and/or hyperuricemia were not mentioned as exclusion criteria, previous/current use of urate-lowering therapies (ULT) and/or colchicine as well as specific dietary patterns were not reported either. None of the 6 studies reported SUA values at baseline and end of follow up. No details about previous history of hyperuricemia or gout in the enrolled patients were provided by any study. Four studies (23–26) reported an increase of SUA in BA-treated patients, however the Authors did not specify whether this increase was classified as hyperuricemia. One study reported no SUA increase over the study period (22). Two studies reported 0 cases of gout among 45 and 60 BA-treated patients at 4 W and 12 W respectively (17, 18). One study reported 1 case of gout in 28 BA-treated patients (3%) and 0 cases in 21 patients receiving placebo for 8 W (26). One study mentioned gout among other causes of BA discontinuation but without further details (25).

## 4. Discussion

BA is a promising LLT to be used alone or as combination therapy in statin-INT and statin-IR patients, however its SUA-increasing effect as well as its putative effect on gout raised safety concerns (27–29). However, previous SLRs were conducted before the publication of the large CLEAR Outcome study and the Authors identified the relatively low patient number as a limitation to derive meaningful conclusions. Furthermore, they highlighted a possible selection bias related to baseline CV risk and renal function. We demonstrated that currently available data from phase 2 and phase 3 RCTs do not allow to confirm a clear association between BA and gout and that several aspects remain unclear. In particular, the paucity of information about SUA levels at baseline and/or at end of follow-up do not allow to quantify the effect sizes for BA-induced SUA elevation. As stated in the 2021 ESC guidelines on CV disease prevention in clinical practice, the achievement of recommended LDL-c goals in patients with high and very high CV risk is of paramount importance (30). Although statins are overall safe and efficacious, muscle side effects severely impact on treatment adherence and retention rate, and a considerable number of patients may not achieve the treatment goal despite the maximum tolerated statin dose. Therefore, combination therapy or switch to other LLTs or is required for an optimal lipid management. The combination of statins and ezetimibe, two oral and relatively low-cost drugs, proved to be adequate in patients with low to moderate risk but still at least one-third patients with high or very high CV risk will require the addition of a third compounds (31). PCSK9 inhibitors revolutionized lipid management but their elevated cost may be a barrier to large-scale use in particular clinical setting (4). Therefore, novel efficacious and less expensive oral therapies are eagerly awaited. In phase 3 RCTs BA addition to background LLT in statin-IR and statin-INT was superior to placebo and allowed to achieve both a more pronounced reduction of LDL-c (14–18) and a better prevention of CV events even in patients at high/very high CV risk (13). Nonetheless, safety data related to its ability to increase SUA levels/trigger gout are still a matter of debate and may limit its implementation in clinical practice. The clinical relevance of SUA goes well beyond the simple association with gout and/or nephrolithiasis (32). In fact, hyperuricemia (with or without gout) is in an independent CV risk factor and based on the association between SUA levels and mortality (both CV and all-cause) and CV events, it is conceivable that CV damage begins with levels of SUA lower than the generally accepted threshold of 6 mg/dl. The Uric Acid Right for Heart Health (URRAH) study identified a SUA threshold value of 4.7 mg/dl for all-cause mortality and 5.6 mg/dl for CV mortality (10). In addition, SUA is an independent risk factor for cerebrovascular events after adjusting for potential confounding variables, including arterial hypertension, and a valid prognostic cut-off value (>4.79 mg/dl) has been identified. Concordantly, SUA levels >5.34 mg/dl (sensitivity 52.32, specificity 63.96, *p* < 0.0001) were the univariate prognostic cut-off value for all heart failure, whereas SUA levels >4.89 mg/dl (sensitivity 68.29, specificity 49.11, *p* < 0.0001) for fatal heart failure (11). Our analysis of BA safety data from 6 phase 2 RCTs and 6 phase 3 RCTs highlighted some aspects deserving to be discussed in light of the future use of BA in clinical practice. First of all, despite the well-known pharmacokinetic characteristics of BA, namely the competition of its glucuronide metabolite and UA for renal excretion, baseline SUA levels are not considered as inclusion/exclusion criteria. By looking at the mean (standard deviation) SUA levels provided by two studies (14–17) it becomes clear that people with baseline hyperuricemia have been enrolled. Furthermore, it is not known for how long these patients have had hyperuricemia or whether they ever received/were receiving any ULT at study enrolment. In addition, we should keep in mind that also patients with normal SUA levels at baseline (with or without ongoing ULT) may still have history of hyperuricemia and/or gout. In this regard, only one study provided information on history of gout and only in patients developing gout during the study period rather than in the entire study cohort. They reported that almost half of the patients developing gout during the study period already had a diagnosis of gout, hence these events should have been classified as gout flares rather than new-onset gout (14). In addition, one of the studies providing baseline SUA levels recruited statin-INT patients with high CV risk (14) and the mean SUA values at baseline imply that SUA management in this population was not optimal despite its clear role as CV risk. Another facet of this complex scenario pertains to time of follow up. The only 2 studies where BA effect size on gout was significant are those with the longest follow up. In particular, the effect size was weakly significant at 12 months (19) but became more consistent up to a median follow up of 40 months (13). This further reinforces the importance of an accurate SUA assessment at baseline but also underlines the need of SUA monitoring overtime. In fact, as nicely shown in the OLE of the CLEAR Harmony study, SUA increase reaches its peak within the first 12 W of treatment and then it remains stable until drug withdrawal (19). Therefore, a careful monitoring of SUA levels may allow to optimise SUA management at baseline, adjust SUA management overtime as needed and ultimately prevent flares/new onset of gout. In conclusion, there is still a knowledge gap on whether BA triggers gout and based on existing data it is reasonable to speculate that this effect may be, at least in part, the result of a selection bias not considering SUA levels and history of gout when starting BA treatment. Real-life data may be instrumental to shed some light on this matter and improve the safety profile of BA in statin-INT and statin-IR patients.

## Data Availability

The original contributions presented in the study are included in the article/**Supplementary Material**, further inquiries can be directed to the corresponding author.

## References

[1] Michos ED McEvoy JW Blumenthal RS. Lipid management for the prevention of atherosclerotic cardiovascular disease. N Engl J Med. (2019) 381:1557–67. 10.1056/NEJMra1806939 31618541

[2] Oesterle A Laufs U Liao JK. Pleiotropic effects of statins on the cardiovascular system. Circ Res. (2017) 120:229–43. 10.1161/CIRCRESAHA.116.308537 28057795 PMC5467317

[3] Bytyçi I Penson PE Mikhailidis DP Wong ND Hernandez AV Sahebkar A Prevalence of statin intolerance: a meta-analysis. Eur Heart J. (2022) 43:3213–23. 10.1093/eurheartj/ehac015 35169843 PMC9757867

[4] Blaum C Brunner FJ Goßling A Kröger F Bay B Lorenz T Target populations and treatment cost for bempedoic acid and PCSK9 inhibitors: a simulation study in a contemporary CAD cohort. Clin Ther. (2021) 43:1583–600. 10.1016/j.clinthera.2021.07.019 34462126

[5] Cicero AFG Fogacci F Cincione I. Evaluating pharmacokinetics of bempedoic acid in the treatment of hypercholesterolemia. Expert Opin Drug Metab Toxicol. (2021) 17(9):1031–8. 10.1080/17425255.2021.1951222 34197267

[6] Pinkosky SL Newton RS Day EA Ford RJ Lhotak S Austin RC Liver-specific ATP-citrate lyase inhibition by bempedoic acid decreases LDL-C and attenuates atherosclerosis. Nat Commun. (2016) 7:13457. 10.1038/ncomms13457 27892461 PMC5133702

[7] Pinkosky SL Filippov S Srivastava RAK Hanselman JC Bradshaw CD Hurley TR AMP-activated protein kinase and ATP-citrate lyase are two distinct molecular targets for ETC-1002, a novel small molecule regulator of lipid and carbohydrate metabolism. J Lipid Res. (2013) 54:134–51. 10.1194/jlr.M030528 23118444 PMC3520520

[8] Wang X Zhang Y Tan H Wang P Zha X Chong W Efficacy and safety of bempedoic acid for prevention of cardiovascular events and diabetes: a systematic review and meta-analysis. Cardiovasc Diabetol. (2020) 19:128. 10.1186/s12933-020-01101-9 32787939 PMC7425167

[9] Saito Y Tanaka A Node K Kobayashi Y. Uric acid and cardiovascular disease: a clinical review. J Cardiol. (2021) 78:51–7. 10.1016/j.jjcc.2020.12.013 33388217

[10] Virdis A Masi S Casiglia E Tikhonoff V Cicero AFG Ungar A Identification of the uric acid thresholds predicting an increased total and cardiovascular mortality over 20 years. Hypertens. (2020) 75:302–8. 10.1161/HYPERTENSIONAHA.119.13643 31813345

[11] Muiesan ML Salvetti M Virdis A Masi S Casiglia E Tikhonoff V Serum uric acid, predicts heart failure in a large Italian cohort: search for a cut-off value the URic acid right for heArt health study. J Hypertens. (2021) 39:62–9. 10.1097/HJH.0000000000002589 32694342

[12] Mengozzi A Pugliese NR Desideri G Masi S Angeli F Barbagallo CM Serum uric acid predicts all-cause and cardiovascular mortality independently of hypertriglyceridemia in cardiometabolic patients without established CV disease: a sub-analysis of the URic acid right for heArt health (URRAH) study. Metabolites. (2023) 13(2):244. 10.3390/metabo13020244 PMC9959524 36837863

[13] Nissen SE Lincoff AM Brennan D Ray KK Mason D Kastelein JJP Bempedoic acid and cardiovascular outcomes in statin-intolerant patients. N Engl J Med. (2023) 388(15):1353–64. 10.1056/NEJMoa2215024 36876740

[14] Goldberg AC Leiter LA Stroes ESG Baum SJ Hanselman JC Bloedon LT Effect of bempedoic acid vs placebo added to maximally tolerated statins on low-density lipoprotein cholesterol in patients at high risk for cardiovascular disease: the CLEAR wisdom randomized clinical trial. JAMA. (2019) 322:1780–8. 10.1001/jama.2019.16585 31714986 PMC6865290

[15] Laufs U Banach M Mancini GBJ Gaudet D Bloedon LT Sterling LR Efficacy and safety of bempedoic acid in patients with hypercholesterolemia and statin intolerance. J Am Heart Assoc. (2019) 8:e011662. 10.1161/JAHA.118.011662 30922146 PMC6509724

[16] Ray KK Bays HE Catapano AL Lalwani ND Bloedon LT Sterling LR Safety and efficacy of bempedoic acid to reduce LDL cholesterol. N Engl J Med. (2019) 380:1022–32. 10.1056/NEJMoa1803917 30865796

[17] Ballantyne CM Banach M Mancini GBJ Lepor NE Hanselman JC Zhao X Efficacy and safety of bempedoic acid added to ezetimibe in statin-intolerant patients with hypercholesterolemia: a randomized, placebo-controlled study. Atherosclerosis. (2018) 277:195–203. 10.1016/j.atherosclerosis.2018.06.002 29910030

[18] Ballantyne CM Laufs U Ray KK Leiter LA Bays HE Goldberg AC Bempedoic acid plus ezetimibe fixed-dose combination in patients with hypercholesterolemia and high CVD risk treated with maximally tolerated statin therapy. Eur J Prev Cardiol. (2020) 27:593–603. 10.1177/2047487319864671 31357887 PMC7153222

[19] Ballantyne CM Banach M Bays HE Catapano AL Laufs U Stroes ESG Long-term safety and efficacy of bempedoic acid in patients with atherosclerotic cardiovascular disease and/or heterozygous familial hypercholesterolemia (from the CLEAR harmony open-label extension study). Am J Cardiol. (2022) 174:1–11. 10.1016/j.amjcard.2022.03.020 35483979

[20] Rubino J MacDougall DE Sterling LR Kelly SE McKenney JM Lalwani ND. Lipid lowering with bempedoic acid added to a proprotein convertase subtilisin/kexin type 9 inhibitor therapy: a randomized, controlled trial. J Clin Lipidol. (2021) 15:593–601. 10.1016/j.jacl.2021.05.002 34172394

[21] Lalwani ND Hanselman JC MacDougall DE Sterling LR Cramer CT. Complementary low-density lipoprotein-cholesterol lowering and pharmacokinetics of adding bempedoic acid (ETC-1002) to high-dose atorvastatin background therapy in hypercholesterolemic patients: a randomized placebo-controlled trial. J Clin Lipidol. (2019) 13:568–79. 10.1016/j.jacl.2019.05.003 31202641

[22] Bays HE Baum SJ Brinton EA Plutzky J Hanselman JC Teng R Effect of bempedoic acid plus ezetimibe fixed-dose combination vs ezetimibe or placebo on low-density lipoprotein cholesterol in patients with type 2 diabetes and hypercholesterolemia not treated with statins. Am J Prev Cardiol. (2021) 8:100278. 10.1016/j.ajpc.2021.100278 34746903 PMC8550983

[23] Thompson PD Rubino J Janik MJ MacDougall DE McBride SJ Margulies JR Use of ETC-1002 to treat hypercholesterolemia in patients with statin intolerance. J Clin Lipidol. (2015) 9:295–304. 10.1016/j.jacl.2015.03.003 26073387

[24] Gutierrez MJ Rosenberg NL Macdougall DE Hanselman JC Margulies JR Strange P Efficacy and safety of ETC-1002, a novel investigational low-density lipoprotein-cholesterol-lowering therapy for the treatment of patients with hypercholesterolemia and type 2 diabetes mellitus. Arterioscler Thromb Vasc Biol. (2014) 34:676–83. 10.1161/ATVBAHA.113.302677 24385236

[25] Ballantyne CM Davidson MH Macdougall DE Bays HE Dicarlo LA Rosenberg NL Efficacy and safety of a novel dual modulator of adenosine triphosphate-citrate lyase and adenosine monophosphate-activated protein kinase in patients with hypercholesterolemia: results of a multicenter, randomized, double-blind, placebo-controlled, parallel-group trial. J Am Coll Cardiol. (2013) 62:1154–62. 10.1016/j.jacc.2013.05.050 23770179

[26] Rubino J MacDougall DE Sterling LR Hanselman JC Nicholls SJ. Combination of bempedoic acid, ezetimibe, and atorvastatin in patients with hypercholesterolemia: a randomized clinical trial. Atherosclerosis. (2021) 320:122–8. 10.1016/j.atherosclerosis.2020.12.023 33514449

[27] Di Minno A Lupoli R Calcaterra I Poggio P Forte F Spadarella G Efficacy and safety of bempedoic acid in patients with hypercholesterolemia: systematic review and meta-analysis of randomised controlled trials. J Am Heart Assoc. (2020) 9:e016262. 10.1161/JAHA.119.016262 32689862 PMC7792250

[28] Cicero AFG Pontremoli R Fogacci F Viazzi F Borghi C. Effect of bempedoic acid on serum uric acid and related outcomes: a systematic review and meta-analysis of the available phase 2 and phase 3 clinical studies. Drug Saf. (2020) 43:727–36. 10.1007/s40264-020-00931-6 32358698

[29] Lin Y Parco C Karathanos A Krieger T Schulze V Chernyak N Clinical efficacy and safety outcomes of bempedoic acid for LDL-c lowering therapy in patients at high cardiovascular risk: a systematic review and meta-analysis. BMJ Open. (2022) 12:e048893. 10.1136/bmjopen-2021-048893 35210334 PMC8883220

[30] Visseren FLJ Mach F Smulders YM Carballo D Koskinas KC Bäck M 2021 ESC guidelines on cardiovascular disease prevention in clinical practice. Eur Heart J. (2021) 42:3227–337. 10.1093/eurheartj/ehab484 34458905

[31] Allahyari A Jernberg T Hagström E Leosdottir M Lundman P Ueda P. Application of the 2019 ESC/EAS dyslipidaemia guidelines to nationwide data of patients with a recent myocardial infarction: a simulation study. Eur Heart J. (2020) 41:3900–9. 10.1093/eurheartj/ehaa034 32072178 PMC7654933

[32] Feig DI Kang D-H Johnson RJ. Uric acid and cardiovascular risk. N Engl J Med. (2008) 359:1811–21. 10.1056/NEJMra0800885 18946066 PMC2684330

