# Supplementary material for: Untangling the relationship between bempedoic acid and gout: results from a systematic literature review
Source: Front Cardiovasc Med. 2023 Oct 25;10:1234601. doi: 10.3389/fcvm.2023.1234601 (PMC10634504; doi:10.3389/fcvm.2023.1234601)
Supplement: Supplementary file 1 [file Datasheet1.docx]

Supplementary Material

Untangling the relationship between bempedoic acid and gout: results from a systematic literature review

**Alessia Alunno^1^*, Francesco Carubbi^1^*, Elena Campanozzi^1^, Federico Bellisario^1^, Jan W Schoones^2^, Francesco Maria Mariani^1^, Evy di Ruscio^1^, Piera Altieri^1^, Claudio Ferri^1^**

^1^University of L’Aquila, Department of Life, Health & Environmental Sciences, Internal Medicine and Nephrology Division, ASL1 Avezzano-Sulmona-L'Aquila, San Salvatore Hospital, L'Aquila, Italy

^2^Directorate of Research Policy, Leiden University Medical Center, Leiden, The Netherlands

*These authors share first authorship

**Correspondence:**Alessia Alunno MD PhD, University of L’Aquila, Department of Life, Health & Environmental Sciences, Internal Medicine and Nephrology Division, ASL1 Avezzano-Sulmona-L'Aquila, San Salvatore Hospital, L'Aquila, Italy

Supplementary appendix for the manuscript

**Supplementary Table S1.** Preferred Reporting Items for Systematic Reviews and Meta-analyses (PRISMA) checklist (from Page MJ et al. The PRISMA 2020 statement: an updated guideline for reporting systematic reviews. BMJ. 2021 Mar 29;372:n71

| **Section and Topic** | **Item #** | **Checklist item** | **Location where item is reported** |
| --- | --- | --- | --- |
| **TITLE** | | |  |
| Title | 1 | Identify the report as a systematic review. | Page 1 |
| **ABSTRACT** | | |  |
| Abstract | 2 | See the PRISMA 2020 for Abstracts checklist. | Page 3 |
| **INTRODUCTION** | | |  |
| Rationale | 3 | Describe the rationale for the review in the context of existing knowledge. | Page 4 |
| Objectives | 4 | Provide an explicit statement of the objective(s) or question(s) the review addresses. | Page 4-5 |
| **METHODS** | | |  |
| Eligibility criteria | 5 | Specify the inclusion and exclusion criteria for the review and how studies were grouped for the syntheses. | Page 4-5 |
| Information sources | 6 | Specify all databases, registers, websites, organisations, reference lists and other sources searched or consulted to identify studies. Specify the date when each source was last searched or consulted. | Page 4-5 |
| Search strategy | 7 | Present the full search strategies for all databases, registers and websites, including any filters and limits used. | Page 4-5 |
| Selection process | 8 | Specify the methods used to decide whether a study met the inclusion criteria of the review, including how many reviewers screened each record and each report retrieved, whether they worked independently, and if applicable, details of automation tools used in the process. | Page 5 |
| Data collection process | 9 | Specify the methods used to collect data from reports, including how many reviewers collected data from each report, whether they worked independently, any processes for obtaining or confirming data from study investigators, and if applicable, details of automation tools used in the process. | Page 5 |
| Data items | 10a | List and define all outcomes for which data were sought. Specify whether all results that were compatible with each outcome domain in each study were sought (e.g. for all measures, time points, analyses), and if not, the methods used to decide which results to collect. | Page 5 |
|  | 10b | List and define all other variables for which data were sought (e.g. participant and intervention characteristics, funding sources). Describe any assumptions made about any missing or unclear information. | Page 5 |
| Study risk of bias assessment | 11 | Specify the methods used to assess risk of bias in the included studies, including details of the tool(s) used, how many reviewers assessed each study and whether they worked independently, and if applicable, details of automation tools used in the process. | Page 5 |
| Effect measures | 12 | Specify for each outcome the effect measure(s) (e.g. risk ratio, mean difference) used in the synthesis or presentation of results. | Page 5 |
| Synthesis methods | 13a | Describe the processes used to decide which studies were eligible for each synthesis (e.g. tabulating the study intervention characteristics and comparing against the planned groups for each synthesis (item #5)). | Page 5 |
|  | 13b | Describe any methods required to prepare the data for presentation or synthesis, such as handling of missing summary statistics, or data conversions. | Page 5 |
|  | 13c | Describe any methods used to tabulate or visually display results of individual studies and syntheses. | Page 5 |
|  | 13d | Describe any methods used to synthesize results and provide a rationale for the choice(s). If meta-analysis was performed, describe the model(s), method(s) to identify the presence and extent of statistical heterogeneity, and software package(s) used. | Page 5 |
|  | 13e | Describe any methods used to explore possible causes of heterogeneity among study results (e.g. subgroup analysis, meta-regression). | Not applicable |
|  | 13f | Describe any sensitivity analyses conducted to assess robustness of the synthesized results. | Not applicable |
| Reporting bias assessment | 14 | Describe any methods used to assess risk of bias due to missing results in a synthesis (arising from reporting biases). | Not applicable |
| Certainty assessment | 15 | Describe any methods used to assess certainty (or confidence) in the body of evidence for an outcome. | Not applicable |
| **RESULTS** | | |  |
| Study selection | 16a | Describe the results of the search and selection process, from the number of records identified in the search to the number of studies included in the review, ideally using a flow diagram. | eFigure1 |
|  | 16b | Cite studies that might appear to meet the inclusion criteria, but which were excluded, and explain why they were excluded. | eFigure1 |
| Study characteristics | 17 | Cite each included study and present its characteristics. | Page 5 |
| Risk of bias in studies | 18 | Present assessments of risk of bias for each included study. | Not applicable |
| Results of individual studies | 19 | For all outcomes, present, for each study: (a) summary statistics for each group (where appropriate) and (b) an effect estimate and its precision (e.g. confidence/credible interval), ideally using structured tables or plots. | eTable2 and 3, Table 1 and 2 |
| Results of syntheses | 20a | For each synthesis, briefly summarise the characteristics and risk of bias among contributing studies. | Not applicable |
|  | 20b | Present results of all statistical syntheses conducted. If meta-analysis was done, present for each the summary estimate and its precision (e.g. confidence/credible interval) and measures of statistical heterogeneity. If comparing groups, describe the direction of the effect. | Page 5-7 |
|  | 20c | Present results of all investigations of possible causes of heterogeneity among study results. | Page 5-7 |
|  | 20d | Present results of all sensitivity analyses conducted to assess the robustness of the synthesized results. | Not applicable |
| Reporting biases | 21 | Present assessments of risk of bias due to missing results (arising from reporting biases) for each synthesis assessed. | Not applicable |
| Certainty of evidence | 22 | Present assessments of certainty (or confidence) in the body of evidence for each outcome assessed. | Not applicable |
| **DISCUSSION** | | |  |
| Discussion | 23a | Provide a general interpretation of the results in the context of other evidence. | Page 8 |
|  | 23b | Discuss any limitations of the evidence included in the review. | Page 8 |
|  | 23c | Discuss any limitations of the review processes used. | Page 10 |
|  | 23d | Discuss implications of the results for practice, policy, and future research. | Page 10 |
| **OTHER INFORMATION** | | |  |
| Registration and protocol | 24a | Provide registration information for the review, including register name and registration number, or state that the review was not registered. | Not applicable |
|  | 24b | Indicate where the review protocol can be accessed, or state that a protocol was not prepared. | A protocol was not prepared |
|  | 24c | Describe and explain any amendments to information provided at registration or in the protocol. | Not applicable |
| Support | 25 | Describe sources of financial or non-financial support for the review, and the role of the funders or sponsors in the review. | No funding |
| Competing interests | 26 | Declare any competing interests of review authors. | No competing interests |
| Availability of data, code and other materials | 27 | Report which of the following are publicly available and where they can be found: template data collection forms; data extracted from included studies; data used for all analyses; analytic code; any other materials used in the review. | template data collection forms, data extracted from included studies, data used for all analyses are available upon request |

*From:*  Page MJ, McKenzie JE, Bossuyt PM, Boutron I, Hoffmann TC, Mulrow CD, et al. The PRISMA 2020 statement: an updated guideline for reporting systematic reviews. BMJ 2021;372:n71. doi: 10.1136/bmj.n71

**Supplementary Text S1. Search strategy**

**PubMed**

<http://www.ncbi.nlm.nih.gov/pubmed?otool=leiden>

(("8-hydroxy-2,2,14,14-tetramethylpentadecanedioic acid"[Supplementary Concept] OR "8-hydroxy-2,2,14,14-tetramethylpentadecanedioic acid"[tw] OR "bempedoic acid"[tw] OR "bempedoic acid"[title/abstract:~2] OR "bempedoic acid*"[tw] OR "nilemdo"[tw] OR "nexletol"[tw] OR "ETC-1002"[tw] OR "ESP-55016"[tw]) AND ("Uric Acid"[Mesh] OR "Uric Acid"[tw] OR "Uric Acid"[title/abstract:~2] OR "Uric Acid*"[tw] OR "Trioxopurine"[tw] OR "Urate"[tw] OR "2,6,8-Trihydroxypurine"[tw] OR "Hyperuricemia"[Mesh] OR "Hyperuricemia"[tw] OR "Hyperuricaemia"[tw] OR "Hyperuricem*"[tw] OR "Hyperuricaem*"[tw] OR "Gout"[Mesh] OR "Gout"[tw] OR "Gouty"[tw] OR "Gout*"[tw] OR "Drug-Related Side Effects and Adverse Reactions"[Mesh] OR "adverse effects"[subheading] OR "adverse"[tw] OR "Side Effects"[tw] OR "Side Effect"[tw] OR "Safety"[Mesh] OR "safety"[tw] OR "safe"[tw] OR "unsafe"[tw]))

**Embase**

<http://ovidsp.ovid.com/ovidweb.cgi?T=JS&PAGE=main&MODE=ovid&D=oemezd>

((*"bempedoic acid"/ OR "8-hydroxy-2,2,14,14-tetramethylpentadecanedioic acid".ti,ab OR "bempedoic acid".ti,ab OR ("bempedoic" ADJ2 "acid").ti,ab OR "bempedoic acid*".ti,ab OR "nilemdo".ti,ab OR "nexletol".ti,ab OR "ETC-1002".ti,ab OR "ESP-55016".ti,ab) AND (exp *"Uric Acid"/ OR exp *"uric acid derivative"/ OR "Uric Acid".ti,ab OR ("Uric"ADJ2 "Acid").ti,ab OR "Uric Acid*".ti,ab OR "Trioxopurine".ti,ab OR "Urate".ti,ab OR "2,6,8-Trihydroxypurine".ti,ab OR exp *"Hyperuricemia"/ OR "Hyperuricemia".ti,ab OR "Hyperuricaemia".ti,ab OR "Hyperuricem*".ti,ab OR "Hyperuricaem*".ti,ab OR exp *"Gout"/ OR "Gout".ti,ab OR "Gouty".ti,ab OR "Gout*".ti,ab OR exp *"adverse drug reaction"/ OR *"bempedoic acid"/ae OR "adverse".ti,ab OR "Side Effects".ti,ab OR "Side Effect".ti,ab OR exp *"Safety"/ OR "safety".ti,ab OR "safe".ti,ab OR "unsafe".ti,ab))

- - NOT conference review.pt
  - NOT (conference review or conference abstract).pt
  - AND (conference abstract).pt

**Web of Science**

<http://isiknowledge.com/wos>

((TI=("bempedoic acid" OR "8 hydroxy 2,2,14,14 tetramethylpentadecanedioic acid" OR "bempedoic acid" OR ("bempedoic" NEAR/2 "acid") OR "bempedoic acid*" OR "nilemdo" OR "nexletol" OR "ETC 1002" OR "ESP 55016") OR AK=("bempedoic acid" OR "8 hydroxy 2,2,14,14 tetramethylpentadecanedioic acid" OR "bempedoic acid" OR ("bempedoic" NEAR/2 "acid") OR "bempedoic acid*" OR "nilemdo" OR "nexletol" OR "ETC 1002" OR "ESP 55016") OR AB=("bempedoic acid" OR "8 hydroxy 2,2,14,14 tetramethylpentadecanedioic acid" OR "bempedoic acid" OR ("bempedoic" NEAR/2 "acid") OR "bempedoic acid*" OR "nilemdo" OR "nexletol" OR "ETC 1002" OR "ESP 55016")) AND (TI=("Uric Acid" OR "uric acid derivative" OR "Uric Acid" OR ("Uric" NEAR/2 "Acid") OR "Uric Acid*" OR "Trioxopurine" OR "Urate" OR "2,6,8 Trihydroxypurine" OR "Hyperuricemia" OR "Hyperuricemia" OR "Hyperuricaemia" OR "Hyperuricem*" OR "Hyperuricaem*" OR "Gout" OR "Gout" OR "Gouty" OR "Gout*" OR "adverse" OR "Side Effects" OR "Side Effect" OR "safety" OR "safe" OR "unsafe") OR AK=("Uric Acid" OR "uric acid derivative" OR "Uric Acid" OR ("Uric" NEAR/2 "Acid") OR "Uric Acid*" OR "Trioxopurine" OR "Urate" OR "2,6,8 Trihydroxypurine" OR "Hyperuricemia" OR "Hyperuricemia" OR "Hyperuricaemia" OR "Hyperuricem*" OR "Hyperuricaem*" OR "Gout" OR "Gout" OR "Gouty" OR "Gout*" OR "adverse" OR "Side Effects" OR "Side Effect" OR "safety" OR "safe" OR "unsafe") OR AB=("Uric Acid" OR "uric acid derivative" OR "Uric Acid" OR ("Uric" NEAR/2 "Acid") OR "Uric Acid*" OR "Trioxopurine" OR "Urate" OR "2,6,8 Trihydroxypurine" OR "Hyperuricemia" OR "Hyperuricemia" OR "Hyperuricaemia" OR "Hyperuricem*" OR "Hyperuricaem*" OR "Gout" OR "Gout" OR "Gouty" OR "Gout*" OR "adverse" OR "Side Effects" OR "Side Effect" OR "safety" OR "safe" OR "unsafe")))

**Cochrane**

<https://www.cochranelibrary.com/advanced-search/search-manager>

(("bempedoic acid" OR "8 hydroxy 2,2,14,14 tetramethylpentadecanedioic acid" OR "bempedoic acid" OR ("bempedoic" NEAR/2 "acid") OR "bempedoic acid*" OR "nilemdo" OR "nexletol" OR "ETC 1002" OR "ESP 55016") AND ("Uric Acid" OR "uric acid derivative" OR "Uric Acid" OR ("Uric" NEAR/2 "Acid") OR "Uric Acid*" OR "Trioxopurine" OR "Urate" OR "2,6,8 Trihydroxypurine" OR "Hyperuricemia" OR "Hyperuricemia" OR "Hyperuricaemia" OR "Hyperuricem*" OR "Hyperuricaem*" OR "Gout" OR "Gout" OR "Gouty" OR "Gout*" OR "adverse" OR "Side Effects" OR "Side Effect" OR "safety" OR "safe" OR "unsafe")):ti,ab,kw

(conference abstract OR meeting abstract OR conference proceeding OR conference proceedings):pt

**Emcare** <http://ovidsp.ovid.com/ovidweb.cgi?T=JS&NEWS=n&CSC=Y&PAGE=main&D=emcr>

(("bempedoic acid"/ OR "8-hydroxy-2,2,14,14-tetramethylpentadecanedioic acid".mp OR "bempedoic acid".mp OR ("bempedoic" ADJ2 "acid").ti,ab OR "bempedoic acid*".mp OR "nilemdo".mp OR "nexletol".mp OR "ETC-1002".mp OR "ESP-55016".mp) AND (exp "Uric Acid"/ OR exp "uric acid derivative"/ OR "Uric Acid".mp OR ("Uric"ADJ2 "Acid").ti,ab OR "Uric Acid*".mp OR "Trioxopurine".mp OR "Urate".mp OR "2,6,8-Trihydroxypurine".mp OR exp "Hyperuricemia"/ OR "Hyperuricemia".mp OR "Hyperuricaemia".mp OR "Hyperuricem*".mp OR "Hyperuricaem*".mp OR exp "Gout"/ OR "Gout".mp OR "Gouty".mp OR "Gout*".mp OR exp *"adverse drug reaction"/ OR "adverse".ti,ab OR "Side Effects".ti,ab OR "Side Effect".ti,ab OR exp *"Safety"/ OR "safety".ti,ab OR "safe".ti,ab OR "unsafe".ti,ab))

**Academic Search Premier**

<http://search.ebscohost.com/login.aspx?authtype=ip,uid&profile=lumc&defaultdb=aph>

Limit to Academic Journals

(TI("bempedoic acid" OR "8 hydroxy 2,2,14,14 tetramethylpentadecanedioic acid" OR "bempedoic acid" OR ("bempedoic" NEAR/2 "acid") OR "bempedoic acid*" OR "nilemdo" OR "nexletol" OR "ETC 1002" OR "ESP 55016") AND TX("Uric Acid" OR "uric acid derivative" OR "Uric Acid" OR ("Uric" NEAR/2 "Acid") OR "Uric Acid*" OR "Trioxopurine" OR "Urate" OR "2,6,8 Trihydroxypurine" OR "Hyperuricemia" OR "Hyperuricemia" OR "Hyperuricaemia" OR "Hyperuricem*" OR "Hyperuricaem*" OR "Gout" OR "Gout" OR "Gouty" OR "Gout*" OR "adverse" OR "Side Effects" OR "Side Effect" OR "safety" OR "safe" OR "unsafe"))

**Google Scholar**

<http://scholar.google.com/>

"bempedoic acid"|"nilemdo"|"nexletol"|"ETC 1002"|"ESP 55016" "Uric Acid"|"Trioxopurine"|"Urate"|"Hyperuricemia"|"Hyperuricaemia" |"Gout"|"Gouty"

**-> 12**

"bempedoic acid"|"nilemdo"|"nexletol"|"ETC 1002"|"ESP 55016 "adverse"|"Side Effects"|"Side Effect"|"safety"|"safe"|"unsafe"

-> 23

**Supplementary Figure S1.** Flowchart of the literature search

**Supplementary Table S2.** Main data of phase 3 randomised controlled trials investigating bempedoic acid

| **STUDY**  **YEAR** | **FOLLOW UP (W)** | **INCLUSION CRITERIA** | | **INTERVENTION**  **N patients**  --------------------------------------------------  **COMPARATOR**  **N patients** | **PRIMARY ENDPOINT (PE)**  **PE MET YES/NO** |
| --- | --- | --- | --- | --- | --- |
|  |  | **Clinical** | **Fasting LDL-c** |  |  |
| CLEAR TRANQUILITY  (2018) | 12 | Statin intolerance* | ≥100mg/dl at screening | BA 180mg + EZE 10mg ±LDS N=181  ------------------------------------  PBO+ EZE 10mg ±LDS N=88 | % Δ LDL-c from baseline at 12W  YES |
| CLEAR SERENITY (2019) | 24 | Primary or secondary CV prevention a nd statin intolerance* | ≥130mg/dl at screening (≥100 if HFH) | BA 180mg ± LDS N=234  ------------------------------------  PBO±LDS N=111 | % Δ LDL-c from baseline at 12W  YES |
| CLEAR HARMONY  (2019) | 52 | ASCVD or HFH or both | ≥70mg/dl despite MTST | BA 180mg + MTST N=1488  ------------------------------------  PBO + MTST N=742 | % Δ LDL-c from baseline at 12W  YES |
| CLEAR WISDOM (2019) | 52 | ASCVD or HFH or both | ≥100mg/dl at screening (≥70mg/dl at recruitment) despite MTST | BA 180mg + MTST N=522  ------------------------------------  PBO + MTST N=257 | % Δ LDL-c from baseline at 12W  YES |
| NCT03337308  (2019) | 12 | ASCVD or HFH or multiple CV risk factors | ≥130 mg/dL (≥100mg/dl if ASCVD and/or HFH) despite MTST | BA 180 mg + EZE 10mg + MTST N=108  -----------------------  BA 180 mg + MTST N=110  EZE 10 mg + MTST N=109  PBO + MTST N=55 | % Δ LDL-c from baseline at 12W  YES |
| CLEAR OUTCOMES  (2023) | 240 | Primary or secondary CV prevention and statin intolerance§ | ≥100 mg/dL | BA 180mg OD ±LDS N=6992  ------------------------------------  PBO±LDS N=6978 | Four-component composite of major adverse CV events, as assessed in a time-to-first-event analysis  YES |
| ASCVD: atherosclerotic cardiovascular disease; HFH: heterozygous familial hypercholesterolemia, CV, cardiovascular; LDL-c, low density lipoprotein cholesterol; MTST: Maximally tolerated statin therapy was defined as the highest intensity statin regimen that a patient was able to maintain, as determined by the investigator, alone or in combination with other lipid-lowering therapies except proprotein convertase subtilisin/kexin 9 (PCSK9) inhibitors, simvastatin >40mg/day and gemfibrozil; BA, bempedoic acid; EZE, ezetimibe; PBO, placebo  *receiving no statin or low-dose statin (LDS) alone or in combination with other lipid-lowering therapies except proprotein convertase subtilisin/kexin 9 (PCSK9) inhibitors, and gemfibrozil and requiring additional LDL-C lowering; §receiving no statin or low-dose statin (LDS) alone or in combination with other lipid-lowering therapies including PCSK9 inhibitors. | | | | | |

**Supplementary Table S3.** Main data of phase 2 randomised controlled trials investigating bempedoic acid and reporting on serum uric acid levels and gout

| **Author, Year**  **RCT N, ref** | **FOLLOW UP (W)** | **INCLUSION CRITERIA** | | **Other LLT before study start** | **INTERVENTION**  **N patients**  ------------------------------------  **COMPARATOR**  **N patients** | **PRIMARY ENDPOINT** |
| --- | --- | --- | --- | --- | --- | --- |
|  |  | **Clinical** | **Laboratory** |  |  |  |
| Ballantyne CM, 2013  NCT01262638 | 12 | BMI 18-35 kg/m2 | LDL-c 130-220 mg/dl  TG<400 | 6W washout | BA 40 mg N=45  BA 80 mg N=44  BA 120 mg N=44  ------------------------------------  PBO N=44 | % Δ LDL-c from baseline at 12W |
| Gutierrez MJ, 2014  NCT01607294 | 4 | T2DM | LDL-c ≥100 mg/dL | 6W washout | BA 80 mg➝ 120mg N=30  ------------------------------------  PBO N=30 | % Δ LDL-c from baseline at 4W |
| Thompson PD, 2015  NCT01751984 | 8 | Statin intolerance  BMI 18-40 kg/m2 | LDL-c 100-220 mg/dL and TG< 350 mg/dL (despite statin)  LDL-c 115-270 mg/dL and TG<400 mg/dL (if statin already discontinued) | 4Wwashout  (if statin ongoing) | BA 60mg ➝ 120mg➝ 180mg➝ 240 mg N=37  ------------------------------------  PBO N=19 | % Δ LDL-c from baseline at 8W |
| Lalwani ND, 2019  NCT02659397 | 4 | BMI 18-40 kg/m2 | Fasting LDL-c 100-220 mg/dL for patients on daily high-intensity statin therapy  Fasting LDL-c 115-220 mg/dL for patients on daily moderate-intensity or low-intensity statin therapy  and  TG<400 mg/dl | 4W washout of ongoing LLT while taking OL atorvastatin 80mg/day | BA 180mg N=45  ------------------------------------  PBO N=23 | % Δ LDL-c from baseline at 4W |
| Bays HE, 2021  NCT03531905 | 12 | T2DM  HbA_1c_ 7-10%  Stable T2DM therapy for ≥ 3M | LDL-c > 70 mg/dL at screening and 100–220 mg/dL after LLT washout | 5W washout | BA 180mg +EZE 10mg N=60  ----------------------------  EZE 10mg N=60  PBO N=59 | % Δ LDL-c from baseline at 12W |
| Rubino J, 2021  NCT03193047 | 8 | None | Fasting LDL-c ≥160 mg/dL (≥70 mg/dL after evolocumab) | 1.5-M washout of ongoing LLT followed by 3-month evolocumab | BA 180mg N=28  ------------------------------------  PBO N=31 | % Δ LDL-c from baseline at 8W |
| The 3 studies that did not provide any information on SUA and gout are not shown in the Table (Thompson PD et al. J Clin Lipidol 2016;10(3):556-67, Ballantyne CM et al. Am J Cardiol 2016;117(12):1928-33, Rubino J et al. Atherosclerosis 2021;320:122–128).  RCT, randomised controlled trial; W, weeks; BMI, bocy mass index; T2DM, type 2 diabetes mellitus; LDL-c, low density lipoprotein cholesterol; TG, triglycerides; LLT, lipid-lowering therapy; OL, open label; M, month; BA, bempedoic acid; PBO, placebo; EZE, ezetimibe. | | | | | | |
